# Innate immunity glycoprotein gp-340 variants may modulate human susceptibility to dental caries

**DOI:** 10.1186/1471-2334-7-57

**Published:** 2007-06-11

**Authors:** Anette Jonasson, Christer Eriksson, Howard F Jenkinson, Carina Källestål, Ingegerd Johansson, Nicklas Strömberg

**Affiliations:** 1 Department of Odontology/Cariology, Umeå University, SE- 901 87 Umeå, Sweden; 2 Department of Oral and Dental Science, University of Bristol, Bristol, UK; 3 Department of Women's and Children's Health/IMCH, Uppsala University, Sweden

## Abstract

**Background:**

Bacterial adhesion is an important determinant of colonization and infection, including dental caries. The salivary scavenger receptor cysteine-rich glycoprotein gp-340, which mediates adhesion of *Streptococcus mutans *(implicated in caries), harbours three major size variants, designated gp-340 I to III, each specific to an individual saliva. Here we have examined the association of the gp-340 I to III polymorphisms with caries experience and adhesion of *S. mutans*.

**Methods:**

A case-referent study was performed in 12-year-old Swedish children with high (n = 19) or low (n = 19) caries experiences. We measured the gp-340 I to III saliva phenotypes and correlated those with multiple outcome measures for caries experience and saliva adhesion of *S. mutans *using the partial least squares (PLS) multivariate projection technique. In addition, we used traditional statistics and 2-year caries increment to verify the established PLS associations, and bacterial adhesion to purified gp-340 I to III proteins to support possible mechanisms.

**Results:**

All except one subject were typed as gp-340 I to III (10, 23 and 4, respectively). The gp-340 I phenotype correlated positively with caries experience (VIP = 1.37) and saliva adhesion of *S. mutans *Ingbritt (VIP = 1.47). The gp-340 II and III phenotypes tended to behave in the opposite way. Moreover, the gp-340 I phenotype tended to show an increased 2-year caries increment compared to phenotypes II/III. Purified gp-340 I protein mediated markedly higher adhesion of *S. mutans *strains Ingbritt and NG8 and *Lactococcus lactis *expressing AgI/II adhesins (SpaP or PAc) compared to gp-340 II and III proteins. In addition, the gp-340 I protein appeared over represented in subjects positive for Db, an allelic acidic PRP variant associated with caries, and subjects positive for both gp-340 I and Db tended to experience more caries than those negative for both proteins.

**Conclusion:**

Gp-340 I behaves as a caries susceptibility protein.

## Background

Dental caries is one of the most prevalent human infectious diseases with life style and genetic factors modifying disease activity [1-4]. The skewed distribution of caries in Western populations today and its weak association with traditional life style factors, *e.g*. sugar intake and oral hygiene [5], suggest genetic components in caries development. Early arguments for a genetic predisposal came from twin studies [6] and the Vipeholm study [1] showing large individual differences in caries development in spite of similar exposures to sugars.

Dental caries is a mixed species infection caused by an ecological shift from commensal toward cariogenic streptococci [2-4], including *Streptococcus mutans *[3]. Among potential caries susceptibility alleles or proteins are accordingly multiple salivary proteins [7], e.g. salivary agglutinin/gp-340 [8-10] and proline-rich proteins (PRPs) [10-12], involved in oral biofilm formation, tissue homeostasis and immunological surveillance [13-15]. While salivary agglutinin mediates aggregation (clearance) and adhesion (colonization) of *S. mutans *and other streptococci [16-18], PRPs primarily attach commensal streptococci and actinomycetes to teeth [13]. Accordingly, caries resistant subjects coincided with increased adhesion of commensal *Actinomyces *and the highly prevalent allelic PRP variants PRP-1 and PRP-2 [10]. By contrast, caries prone subjects coincided with increased saliva adhesion of *S. mutans *and Db, a low prevalence allelic acidic PRP variant [10]. Salivary agglutinin is the major adhesion and aggregation factor in saliva for *S. mutans *and is targeted by its major surface adhesin polypeptide, antigen I/II (AgI/II) [19]. Oral viridans streptococci generally express conserved, but species-specific, AgI/II polypeptides [19]. However, while the AgI/II adhesin SpaP (or PAc) expressed by *S. mutans *is the principal surface adhesin interacting with gp-340, the commensal organism *Streptococcus gordonii *expresses additional gp-340-interacting adhesins, including Hsa [20,21]. The AgI/II polypeptides interact with host cells and are potent activators of cell-mediated responses [19,22], and have been used for vaccine and anti-adhesion protection against *S. mutans *and dental caries [23,24]. We have shown salivary agglutinin to be identical to the scavenger receptor cysteine-rich glycoprotein gp-340 [9] and found three prevalent size variants of saliva gp-340, designated gp-340 I to III, each specific to individual donors [25]. However, the gp-340 I to III size polymorphisms have not been investigated as relates to susceptibility or resistance to dental caries or to differences in AgI/II-mediated adhesion of *S. mutans*.

Gp-340 [8,9,26] or DMBT1 (deleted in malignant brain tumour, [27]) are protein homologs, encoded by the same *dmbt1 *gene. They are mucin-like multidomain proteins, composed of 14 repeating scavenger receptor cysteine-rich SRCR domains intercalated by SID domains and followed by CUB and ZP domains. In saliva, gp-340 exists as an oligomer complexed with secretory immunoglobulin A (S-IgA) [16,28]. Salivary gp-340/agglutinin aggregates a wide array of bacteria and viruses via O-glycosylated Ser/Thr-rich SID repeats and N-glycans [9,20,29,30]. It behaves differently in fluid versus surface adsorbed form [20,21]. While fluid phase gp-340 aggregates only certain streptococcal phenotypes, surface adsorbed gp-340 selectively adhere other phenotypes (even of the same bacterial species) [20,21]. Moreover, the SRCR, CUB and ZP domains interact with multiple protein ligands [14]: SpD and SpA collectins [31], lactoferrin [32], complement factor C1q [33], S-IgA [28], and with MUC5B [34]. Gp-340/DMBT1 are present on macrophages, in lung and brain tissues and in gastric and intestinal mucosa [26], and activate macrophages [31] and PMN cells as well as affect the differentiation mode of epithelial cells [14]. Accordingly, the gp-340/DMBT1 proteins are considered pattern recognition molecules in various host innate defences [14].

The partial least squares (PLS) multivariate projection technique derives its usefulness from its ability to deal with multiple and noisy variables and multicollinearity in data structures [35,36]. The PLS technique is designed to handle multiple variables measured on relatively few subjects (so-called “short and fat” data structures) compared to traditional statistics that measures a few variables on many subjects (so-called “long and lean” data structures). It has been applied in genomics, proteomics and metabonomics [36], in biochemistry to delineate the chemical features of the RGRPQ peptide derived from the caries resistance PRP-1 polypeptide [37,38], and to delineate biomarkers or other clinical traits in human diseases [36,10,39].

The aim of the present study was to utilize the PLS method to correlate the gp-340 I to III size variants with caries experience and saliva adhesion of *S. mutans *in children with high (n = 19) or low (n = 19) caries experience, as well as to substantiate possible mechanisms behind identified associations. The results reveal a positive association of gp-340 I with both caries experience and saliva adhesion of *S. mutans*, and that purified gp-340 I protein mediates increased AgI/II-mediated adhesion of *S. mutans*.

## Methods

### Study groups, clinical recordings and saliva measurements

Twelve-year-old high caries cases (n = 19) and low caries referents (n = 19) from three Public Dental Health Clinics in Sweden were used in the present study. The 12-year-old children were nested within the northern portion of a Swedish nationwide cohort study of 3,400 children [5,10]. The cases were randomly selected from the children with 4 or more new enamel/dentin lesions during the latest year (mean baseline DMFS = 5.0), and the referents were matched for gender and living area from caries free individuals (baseline DMFS = 0). Caries was recorded at base-line and after 2 years (2-year increment of dentin and enamel lesions). The study was approved by the Ethics Committee at Umeå University, Umeå, Sweden.

Data collection and saliva analyses were largely performed as described [5,10]. Briefly, a questionnaire was used for analyses of life style factors, *e.g*. sugar intake, oral hygiene, fluoride exposure etc, and fresh whole saliva for analyses of saliva factors, *e.g*. flow rate, pH, buffer capacity etc. Parotid saliva, collected on ice using Lashley cups and 3 % citric acid stimulation, was stored frozen (-80°C) in aliquots for subsequent Db and gp-340 phenotyping and the ability to mediate adhesion of *S. mutans *to saliva-coated hydroxyapatite [10]. Typing of Db+ subjects (hetero- or homozygous) versus Db- subjects (completely lacking Db but harbouring two or more of the allelic PRP-1, PRP-2, PIF or Pa variants), used native alkaline electrophoresis as described [10]. Missing saliva data for some measurements resulted in final analyses using either 36, 37 or 38 subjects.

### Gp-340 phenotyping

Gp-340 I to III typing of parotid saliva was done by Western blot using mAb143 directed to the gp-340 protein core [9,25]. Saliva samples were boiled in sample buffer (62.5 mM Tris, 10.1 % glycerol, 2 % SDS, 0.01 % pyronin) for 5 minutes. Proteins were separated by SDS-PAGE using precasted 5 % polyacrylamide gels (Bio-Rad, Hercules, CA) and running buffer (25 mM Tris, 192 mM glycin, 0.1 % SDS), pH 8.3. Separated proteins were transferred to an Immobilon-P transfer membrane (0.45 μm, Millipore, Billerica, MA) using 65 mA/membrane for 60 minutes. Subsequently, the membranes were incubated with 5% non-fat dried milk in TBS-T (50 mM Tris, 150 mM NaCl_2 _and 0.05 % Tween 20), pH 7.4, overnight at 4°C. The blocked membranes were overlaid with mAb143, diluted 1:100,000 in TBS-T with 5 % non-fat dried milk, for one hour at room temperature. After repeated washes with TBS-T, the membranes were incubated for one hour with horseradish peroxidase-conjugated goat anti-mouse IgG (Nordic Biosite, Stockholm, Sweden) in TBS-T containing 5 % non-fat dried milk. After repeated washes, bands were detected using chemiluminescence (SuperSignal Substrate, Pierce, Rockford, IL).

Gp-340 I to III phenotyping of saliva from the 38 children was performed using three gp-340 I to III saliva phenotypes as typing references. The majority of gp-340 I to III phenotypes, including all I and III phenotypes, were distinguished in a single electrophoretic analysis. Some gp-340 II phenotypes required one or two additional electrophoretic runs to safely be distinguished from III. All salivas, except one with a double band character, adhered to the single band and size typing criteria.

### Purification of gp-340 I to III proteins

The gp-340 I to III protein variants were purified from parotid saliva from three donors as described [9,25]. Briefly, fresh parotid saliva diluted 1:1 in 10 mM phosphate buffered saline (PBS, K_2_HPO_4_, 150 mM NaCl), pH 6.8, was mixed with a suspension *S. mutans *Ingbritt (5 × 10^9 ^cells/ml) and allowed to aggregate for 60 minutes at 37°C. After addition of 50 mM EDTA to the pelleted aggregates, released gp-340 was purified by gel filtration (Superdex 200 26/60; Pharmacia, Uppsala, Sweden). Protein concentration and purity of isolated gp-340 I to III proteins were determined by the DC protein assay (Bio-Rad) with bovine serum albumin (BSA) as a standard, densitometric analyses of Coomassie Blue-stained gels and by Western blotting with mAb143.

### Bacterial strains and culturing

*S. mutans *strains Ingbritt, NG8 and mutant 834 were grown in Brain Heart Infusion broth (BHI; Difco laboratories, Detroit, MI) or Jordan broth [20] at 37°C for 14–16 hours. The isogenic *S. mutans *834 Δ *pac *mutant was generated from wild type strain NG8 by allelic replacement [40] and cultured as described above except for addition of erythromycin (5 μg/ml) to the media [20]. The *pac *and *spaP *genes were cloned into the vector pTREX1-usp45LS and expressed on the surface of wild type *Lactococcus lactis *MG1363 as described previously [21]. Lactococci strains were grown in M17 broth (Merck, Darmstadt, Germany) at 30°C for 14–16 hours with or without (wild type MG1363) addition of erythromycin (5 μg/ml) to the media. The bacterial cells were [^35^S]-labelled by adding [^35^S]methionine to the growth medium prior to culturing as described [20].

### Adhesion of bacteria to gp-340 I to III proteins

Bacterial adhesion to hydroxyapatite beads coated with purified gp-340 protein was analysed [20]. Briefly, after hydration of the hydroxyapatite beads (5 mg/well, Macro-Prep ceramic hydroxyapatite Type II, 80 μm, Bio-Rad) in buffered KCl (1 mM KH_2_PO_4_-K_2_HPO_4 _buffer, pH 6.5, containing 50 mM KCl, 1 mM CaCl_2 _and 0.1 mM MgCl_2_) overnight at 4°C, the beads were coated with gp-340 protein (1–6 or 2 μg/ml in buffered KCl) for 60 minutes at room temperature. The beads were blocked with 5 % BSA for 60 minutes, washed, and incubated with [^35^S]methionine-labelled bacteria (5 × 10^8 ^cells/ml in buffered KCl supplemented with 0.5 % BSA) for 60 minutes at room temperature. After washings, the numbers of bound bacteria were measured by scintillation counting.

### PLS modelling

The partial least squares (PLS) projection method was performed using the Simca-P software (version 10.5, Umetrics AB, Umeå, Sweden) as described [35,36]. PLS establishes the information in x variables that relates to the variation in Y in a multivariate model. An X matrix, containing life-style (*e.g*. sugar intake, oral hygiene, use of fluorides) and saliva (*e.g*. allelic PRP variants, pH, buffer capacity, gp-340 I to III) variables were modelled against two different Y matrices. One Y matrix was composed of eight individual caries measures (*i. e*. fillings, and dentine or enamel caries at various tooth surfaces as described) [10], and another Y matrix consisted of saliva adhesion of *S. mutans *(*i. e*. adhesion of *S. mutans *to hydroxyapatite beads coated with parotid saliva diluted 1:1). The X and Y matrices are described in detail elsewhere [10], except for the present inclusion of gp-340 I to III as qualitative variables in the X matrices. The associations between each x-variable and the Y matrix are expressed as PLS regression coefficients and VIP-values (Variable Importance in Projection), where a VIP>1 indicates that the x-variable is influential for explaining Y.

### Statistics

Differences between group means (bacterial adhesion) were tested with Student's unpaired *t *test (2 groups) or ANOVA followed by Tukey's test (>2 groups). Differences in distribution were tested with the Chi^2^-test. Group differences in caries scores (DMFS or caries increment) and gp-340 antibody staining/amounts were tested with the Mann Whitney U test. All tests were 2-sided except for caries increment, and the significance level was set at p < 0.05.

## Results

### The gp-340 I phenotype correlated with susceptibility to caries

**Figure 1:**
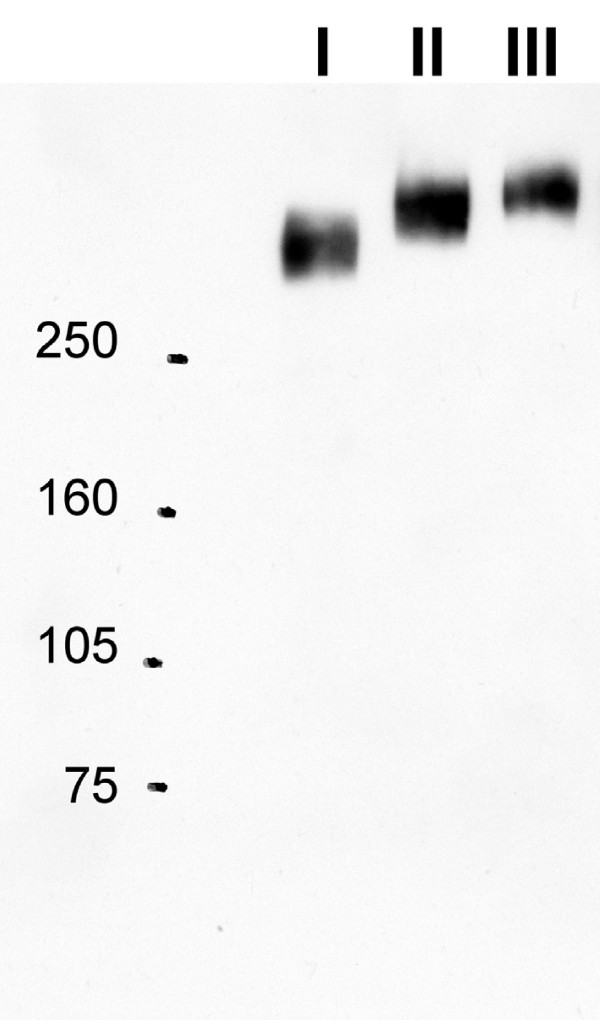
**Saliva gp-340 phenotypes I to III**. Illustration of representative gp-340 I to III saliva phenotypes observed among children upon Western blotting of parotid saliva samples with mAb143. Unreduced parotid saliva samples were separated by SDS-PAGE on 5% gels. Molecular masses (kDa, left) and gp-340 I to III phenotype (top) of the three saliva donors are marked.

The children with high (n = 19) or low caries experience (n = 19) were phenotyped for gp-340 I to III protein variants, based on their saliva gp-340 protein banding pattern by anti-gp-340 mAb143 in Western blot (Figure 1). All subjects except one displayed the gp-340 I, II or III phenotypes (10, 23 and 4, respectively). A single subject with a gp-340 double band character [25] was not considered in the subsequent analyses.

**Figure 2:**
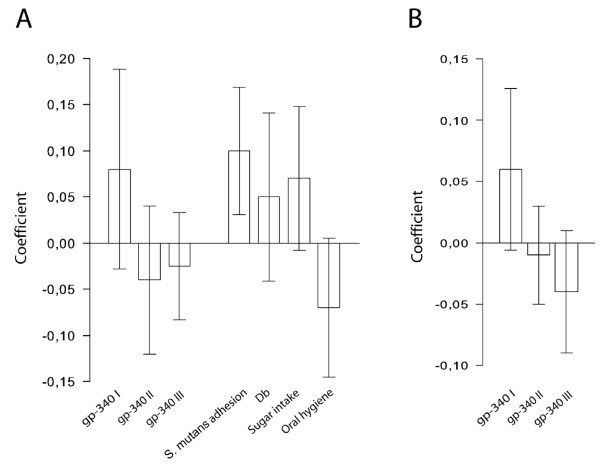
**Association of gp-340 I with caries susceptibility and saliva adhesion of *S. mutans***. PLS correlation coefficients, with 95 % CI, for (A) gp-340 I to III phenotypes and some selected caries-associated variables from modelling with caries experience in the children as dependent variable, and (B) gp-340 I to III phenotypes from modelling with saliva adhesion of *S. mutans *in the children as dependent variable.

In multivariate PLS modelling (including the multiple life style and saliva variables), the gp-340 I to III phenotypes were correlated with caries experience (Figure 2A). The variable set rendered a two component PLS modelexplaining (R^2^) and predicting (Q^2^) the variance in caries experience at an acceptable level (R^2 ^= 0.56, Q^2 ^= 0.20). In this model, the gp-340 I phenotype correlated (VIP = 1.37) with a high caries experience, while the gp-340 II and III phenotypes tended to behave in the opposite way (VIPs<1) (Figure 2A). The correlation between gp-340 I phenotype and caries occurred at a level similar to traditional factors (*e.g*. sugar intake and oral hygiene) and to novel host factors (*e.g*. saliva adhesion of *S. mutans *and the susceptibility protein Db) (Figure 2A). The PLS model was stable, *i.e*. the correlations for the gp-340 I to III phenotypes remained the same when modelling was done with 1/3 of the subjects randomly and consecutively excluded (data not shown).

### The gp-340 I saliva phenotype mediated increased adhesion of *S. mutans *and AgI/II polypeptides

PLS was used to correlate the gp-340 I to III phenotypes with the ability of saliva from the children to mediate adhesion of *S. mutans *to saliva-coated hydroxyapatite (Figure 2B). A one component PLS model with R^2 ^= 0.51 and Q^2 ^= 0.27 was generated. The gp-340 I phenotype coincided with a high adhesion of *S. mutans *Ingbritt (VIP = 1.47), while the opposite tended to be true for gp-340 phenotypes II (VIP<1.0) and III (VIP = 1.06) (Figure 2B).

**Figure 3:**
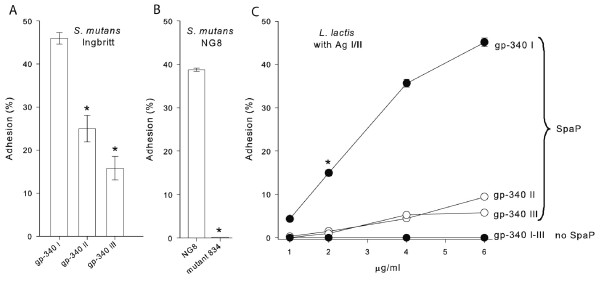
**Association of gp-340 I with increased *S. mutans *adhesion**. (A-C) Bacterial adhesion to purified gp-340 I toIII proteins coated on hydroxyapatite surfaces (2 ug/ml for fixed amounts of protein). Data are presented as mean ± SE from double measurements of repeated experimental runs. (A) Adhesion of *S. mutans *Ingbritt to gp-340 I vs II or III (* p < 0.001, respectively). Similar results were obtained irrespective of culturing in BHI or Jordan broth. (B) Adhesion of *S. mutans *NG8 and isogenic mutant 834, disrupted in the *pac *(AgI/II polypeptide) gene, to gp-340 I protein (* p < 0.001). (C) Adhesion of *L. lactis *expressing SpaP (from *S. mutans*) or vector control (wild-type) *L. lactis *MG1363 to serial dilutions of gp-340 I vs II or III (* p < 0.001, respectively). Similar results were obtained when *L. lactis *expressing PAc was bound to serial dilutions of gp-340 I to III.

We next investigated if purified gp-340 proteins I to III mediated different adhesion levels of *S. mutans *through recognition by AgI/II proteins (Figures 3A to 3C). The gp-340 I protein promoted markedly higher adhesion of *S. mutans *Ingbritt and NG8 compared to gp-340 II and III (Figure 3A, 46%, 25% and 16% adhering cells, respectively, p < 0.001 for I vs II or III). *S. mutans *mutant 834, derived from *S. mutans *NG8 and abrogated in expression of PAc (AgI/II) protein, showed no adhesion to gp-340 I (Figure 3B). Moreover, the gp-340 I protein mediated several fold higher adhesion than gp-340 II and III of *Lactococcus lactis *expressing AgI/II polypeptides from strains Ingbritt and NG8 (SpaP and PAc, respectively) (Figure 3C). Wild-type vector control *L. lactis *MG1363 cells not expressing any AgI/II protein did not adhere to gp-340 proteins I, II or III (Figure 3C). Taken together, these results suggest that gp-340 I is preferentially recognized by the *S. mutans *AgI/II polypeptide and promotes high affinity adhesion and oral colonization by pathogenic *S. mutans *bacteria.

### The gp-340 I phenotype coincides with an increased caries increment and the caries susceptibility protein Db

**Figure 4:**
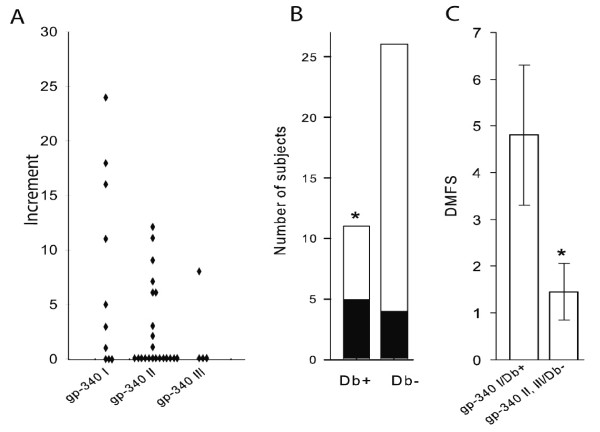
**Association of the gp-340 I phenotype with increased caries increment and the caries susceptibility allele Db**. (A) Plot of the individual 2-year caries increment scores for the gp-340 phenotypes I to III (gp340 I versus II/III, median scores 4 and 0, respectively, p = 0.027). (B) Numbers of gp-340 I positive subjects (■) among Db+ and Db- subjects (* p = 0.051). (C) Caries experience (mean DMFS ± SE) in subjects positive for gp-340 I and Db (gp-340I/Db+) versus subjects negative for both proteins (gp-340II, III/Db-) (* p = 0.023).

To validate our findings from the PLS model (using eight dependent Y caries measures), we also analysed the gp-340 I versus II/III phenotypes for differences in 2-year caries increment by means of traditional statistics (Figure 4A). The caries increment was higher for gp-340 I compared to gp-340 II/III phenotypes (p = 0.027, Figure 4A). In addition, the gp-340 I versus II/III phenotypes did not differ significantly in gp-340 amounts as inferred from mAb143 staining of gp-340 in individual salivas upon Western blotting (data not shown).

We have previously reported that the allelic acid PRP protein variant Db correlates with a high caries experience [10]. The present findings showed the gp-340 I protein to be more common among Db+ subjects (5/11 = 45 %), than among Db- subjects (4/26 = 15%, p = 0.051) harbouring other allelic acidic PRP variants (*i.e*. PRP-1, PRP-2, PIF, Pa) (Figure 4B). The phenotypes positive for both gp-340 I and Db experienced more caries than those negative for both proteins (4.8 versus 1.5 DMFS, respectively, p = 0.023, Figure 4C).

## Discussion

This study suggests for the first time a potential role for gp-340/DMBT1 polymorphisms in human diseases beyond cancer, as it implies the gp-340 I protein as a caries susceptibility protein. Accordingly, the gp-340 I phenotype correlated positively with caries experience when analysed among other variables by PLS modelling, as well as coincided with an increased 2-year caries increment. Moreover, the gp-340 I phenotype correlated positively with saliva adhesion of *S. mutans*, an intermediate caries measure, and purified gp-340 I protein mediated increased adhesion of the same organism and its major AgI/II surface adhesin. Finally, the gp-340 I protein was overrepresented in subjects positive for Db, another caries susceptibility factor.

It is possible that gp-340 I acts as a caries susceptibility protein by increasing the adhesion and colonisation of *S. mutans*. Gp-340 I positive subjects displayed increased saliva adhesion of *S. mutans*, a function previously associated with caries development in the same cohort. Moreover, purified gp-340 I protein enhanced adhesion of both *S. mutans *and lactococci expressing the AgI/II adhesin (SpaP or PAc) from *S. mutans*. The gp-340 protein I may exhibit adhesion epitopes of higher affinity or availability than variants II and III. By contrast, neither lactococci expressing AgI/II nor *S. mutans *discriminated between variants I to III when aggregated, as opposed to when adhered, by the gp-340 size variants [25]. We speculate, although only gp-340 I to III proteins purified from single donors have been tested, that aggregation (by fluid gp-340) and adhesion (by surface adsorbed gp-340) may involve different recognition epitopes for *S. mutans*, and that the surface-associated epitope alone may be affected by the gp-340 I to III polymorphisms. This interpretation is consistent with the generally deviating adhesive behaviour of fluid and surface adsorbed gp-340 toward different streptococcal phenotypes, and that both gp-340 and AgI/II [19,41] are multidomain polypeptides with several potential binding sites. Finally, since many oral viridans streptococci express AgI/II polypeptides interacting with gp-340, the gp-340 protein I may besides *S. mutans *promote colonization of cariogenic phenotypes of additional streptococcal species.

The mucin-like and multifunctional gp-340 I protein variant may differ in a variety of its protein and cellular ligand interactions and, consequently, impair protection against caries by a multiplicity of mechanisms. In this context, it is noteworthy that gp-340 I versus II/III proteins appears to differ in glycosylation [25], and that VNTR associated with cancer are present in gp-340 [42]. Both carbohydrate modifications, involving SID domains with bacterial ligand interactions, and protein modifications, involving SRCR domains with protein and cellular ligand interactions, may accordingly modify the biological properties of gp-340/DMBT1. Salivary MUC5B and MUC7 [43,44] also harbour size variants associated with variations in VNTR and glycosylation. At present, however, we do not know if the gp-340 I to III size variations between subjects occur also in tissues other than saliva. Anyhow, gp-340 is expressed on macrophages and at various oro-gastro-intestinal tissue sites involved in immunological surveillance. We hypothesize that the down stream immunological processing of AgI/II complexes with gp-340 may be impaired in gp-340 I phenotypes. In the case of *S. mutans*, AgI/II is a major adhesin and vaccine candidate and further studies on innate and immune modifying properties of gp-340 polymorphisms may be fruitful.

A role for gp-340 I in susceptibility to caries is consistent with its potential link to Db, a caries susceptibility PRP protein variant or allele. Gp-340 I was over represented in Db+ as compared to Db- subjects, and gp-340 I+/Db+ phenotypes experienced more caries than those negative for both proteins. The gp-340 and PRP scavenger protein families are located on separate chromosomes, 10 and 12, respectively, but may cooperate in adhesion or molecular networking to neutralize non self ligands in saliva. Notably, both gp-340 I and Db correlate positively with saliva adhesion of *S. mutans *[10] and gp-340 interacts with multiple salivary proteins, *e.g*. S-IgA, lactoferrin, SpD and MUC5B, and co-operate with SpD to neutralize influenza virus in saliva [30].

The present work further emphasizes the usefulness of the PLS method to identify potential target molecules for host susceptibility or resistance in small clinical samples. Notably, the potential PRP-1 resistance polypeptide targeted by this approach releases via bacterial proteolysis an RGRPQ peptide affecting key properties of biofilm formation (i. e. adhesion, proliferation and local pH) *in vitro *and *in vivo *[37,38]. Whether the gp-340 size variants are subject to similar proteolytic events or will provide similar drug candidates remains to be determined. Moreover, the potential gp-340 I and Db susceptibility markers are present at about a 15–20% prevalence level. However, it is reasonable to assume that the predictive value of single susceptibility factors – similar to many other potentially polygenetic diseases – will be low for the multifactorial and chronic caries disease [45]. Finally, further studies on host polymorphisms and their evaluation in disease profiling and risk assessment using larger clinical samples may reveal the usefulness of the gp-340 and other host polymorphisms in risk assessment of caries in a clinical setting.

## Conclusion

This report shows that the scavenger protein gp-340 size variant I coincides with host susceptibility to dental caries, and that increased AgI/II-mediated adhesion of the cariogenic bacterium *S. mutans *may be an underlying mechanism.

## Competing interests

The author(s) declare that they have no competing interests.

## Authors' contributions

AJ: planning, adhesion experiments, PLS modeling, data analyses, and together with NS drafting of the manuscript.

CE: gp-340 purification and adhesion experiments.

CK, HFJ, IJ and NS: overall design and planning, co-ordination and writing of the final manuscript.

All authors contributed to writing of the final manuscript.

All authors read and approved the final manuscript.

## Pre-publication history

The pre-publication history for this paper can be accessed here:


